# Attitudes Toward and Usage of Evidence-Based Mental Health Practices for Autistic Youth in Bangladesh and Germany: A Cross-Cultural Comparison

**DOI:** 10.1007/s10803-023-06223-z

**Published:** 2024-01-26

**Authors:** Maleka Pervin, Nina Marie Hansmann, York Hagmayer

**Affiliations:** 1 Institute of Psychology, Georg August University of Göttingen, Göttingen, Germany; 2 Department of Psychology, University of Dhaka, Dhaka, Bangladesh

**Keywords:** Mental health professionals, Attitudes, Evidence-based practice, Autism spectrum disorder

## Abstract

The implementation of evidence-based practices (EBPs) for autistic youth is a critical concern worldwide. Research examining factors facilitating the implementation of EBPs found that providers’ attitudes are an important factor. In this study, we evaluated cross-cultural differences in attitudes toward and use of EBPs. We tested socio-demographic factors as predictors of attitudes, and attitudes as predictors of EBPs use among mental health professionals working with autistic youth in Bangladesh and Germany. We used purposeful sampling. Two-hundred-ninety-two professionals who worked in a clinical setting responded to the survey and fulfilled the inclusion criteria (101 in Bangladesh, 191 in Germany). Participants were asked to respond to nine subscales of the Evidence-Based Practice Attitude Scale-36 (EBPAS-36), to indicate which of nine types of treatments they used, and to provide sociodemographic data. Measurement invariance across countries could be established for four subscales of the EBPAS-36. Comparative analyses of attitudes showed that professionals in both countries were open to using EBPs, but German practitioners were more likely to use EBPs when they appealed to them. By contrast, Bangladeshi professionals claimed to be more likely to adopt an EBP when required and to be more willing to learn EBPs to enhance job security. The relationship between caseload and attitudes varied between countries. A broader variety of EBPs was used in Germany. The findings highlight the importance of considering country-specific factors when implementing EBPs. Directions for conducting comparative studies on mental health professionals’ attitudes towards EBP including methodological considerations are discussed.

**Supplementary Information:**

The online version contains supplementary material available at 10.1007/s10803-023-06223-z.

Autism spectrum disorder (ASD) is a global public health concern because of its prevalence and its effects on individuals and families (Catalano et al., 2018; Hou et al., 2018; Lyall et al., 2017). In recent years, cases of autism have been on the rise. The estimated global prevalence of ASD is 1–2%, but estimates are higher in high-income countries (HIC; Baxter et al., 2015; Elsabbagh et al., 2012). Germany (a HIC) has a low rate of autism of 0.07% or 1 in 139 people (World Population Review, 2021). An earlier study, however, reported rates between 0.22 and 0.38% for the period 2006–2012 based on a sample of 0- to 24-year-olds (Bachmann et al., 2018). The rate is lower than in other HICs. The prevalence rates of ASD in lower middle-income countries (LMICs) and low-income countries are uncertain. In Bangladesh (a LMIC), the observed prevalence ranged from 0.15 to 0.8% (Hossain et al., 2017).

## Healthcare Delivery Services for ASD in Germany and Bangladesh

In Germany, treatments and services for those living with mental disorders are mainly provided by specialized mental health professionals. There are an estimated 14,354 psychiatrists working in a country of 83.29 million people (Melcop et al., 2019; https://www.dgppn.de/Resources/Persistent/17452fbcf559a53a36e71334cde8d18e8d6793fa/20210727_Factsheet_Kennzahlen.pdf). Autism Therapy Centres provide outpatient therapeutic support and treatment (Bundesverband autismus Deutschland e.V., 2020). The Federal Association of Autism Germany currently lists 145 therapy centres throughout Germany (Bundesverband autismus Deutschland e.V., 2022). Social or youth welfare services provide specialized autism therapy for a better integration and participation of patients (Frese, 2021). The multimodal and multi-professional therapy involves psychiatrists, psychotherapists, psychologists, pedagogues, social workers, occupational therapists, and speech therapists (Bundesverband Autismus Deutschland e.V., 2017). Surveys showed that autistic individuals receive a wide variety of different treatments, including behaviour therapy, cognitive behaviour therapy, pharmacotherapy, ergotherapy, and speech therapy (Bachmann & Hoffmann, 2015; Bachmann et al., 2018).

In contrast, only 200 psychiatrists and 50 psychologists are available for roughly 163 million people in Bangladesh (Hasan et al., 2021; WHO, 2011). The National Institute of Mental Health (NIMH) and the Institute of Pediatric Neurodisorders and Autism (IPNA) provide comprehensive services to children with neurodevelopmental disorders (NDDs). In addition, private and non-governmental autism centers and clinics offer a range of services to autistic youth. Special schools provide speech and language therapy, play therapy, sensory integration therapy, music therapy, occupational therapy, and special education services (Situation Assessment of Autism and Neurodevelopmental Disorders in Bangladesh, 2016). A National Steering Committee for Autism and NDD (NSCAND) recommended a phased implementation of a national strategic plan for Neurodevelopmental disorders from 2016 to 2021 to ensure a better quality of life for children with NDDs.

Currently, there are no mandatory guidelines concerning ASD in Bangladesh or Germany. However, in May 2021 an evidence-based guideline "Autism Spectrum Disorders in Childhood, Adolescence, and Adulthood. Part 2: Therapy" was published by the Association of Scientific Medical Societies in Germany (http://www.awmf.org/leitlinien/detail/ll/028-018.html). It provides guidance on the assessment and treatment of ASD. In Bangladesh, every institution follows their own guidelines. Mental health service providers often follow recommendations of guidelines by the National Institute for Health and Care Excellence (NICE) in UK or the American Psychiatric Association (APA, 2013; Ehsan et al., 2018).

## Mental Health Professionals’ Attitudes Towards Evidence-Based Practices

Attitudes are a determinant of the decision of whether to try a new practice and it can impact decision processes regarding innovation (Candel & Pennings, 1999; Frambach & Schillewaert, 2002; Rogers, 1995). In general, attitudes are defined as “a psychological tendency that is expressed by evaluating a particular entity with some degree of favour or disfavour” (Eagly & Chaiken, 1993, p. 1). Attitudes towards evidence-based practices (EBPs)^1^ refer to opinions and perceptions of EBPs and their implementation process (Aarons, 2004).

Several theoretical frameworks have been proposed to explain under which conditions EBPs are implemented in mental health care settings in the way they were designed (cf. Michie et al., 2005). These frameworks identified factors that may influence the adoption of EBP as a general approach to clinical work and the usage of specific EBPs. There are factors on at the level of the individual, the organization, the community, and the system. A recent overview of the major theoretical frameworks summarized individual-level factors (Williams & Beidas, 2019) and identified ten factors shared by the different frameworks. Among them were the attitudes of the professionals.

Research investigated how mental health professionals’ attitudes may facilitate or hinder the implementation of EBPs (Aarons et al., 2012; Greenhalgh et al., 2004; Lilienfeld et al., 2013; Rye et al., 2017; Wisdom et al., 2014). For example, studies building upon the Exploration, Preparation, Implementation, Sustainment (EPIS) framework found that the attitudes of practitioners towards the use of new practices predict their uptake of EBPs (Moullin et al., 2019). Other studies also found that professionals’ attitudes are important determinants of the implementation and use of innovations (Aarons, 2004, 2005; Aarons et al., 2012; Frambach & Schillewaert, 2002).

Attitudes of mental health professionals in turn seem to depend on a multitude of other factors including individual demographic factors (e.g., gender, years of experience) and organizational factors (e.g., leadership, social climate and organizational support, policies, and system factors; Aarons, 2004; Aarons et al., 2010, 2012; Beidas et al., 2015; Connors et al., 2019; Damschroder et al., 2009; Greenhalgh et al., 2004; Locke et al., 2019; Okamura et al., 2018; Powell et al., 2017; Rye et al., 2019; Smith, 2013; Vassos & Carroll, 2016; Wisdom et al., 2014). For example, in some studies men reported more negative attitudes towards adopting EBPs than females, whereas other studies found no sex differences (Aarons et al., 2012; Egeland et al., 2016; Rye et al., 2019; van Sonsbeek et al., 2015). Private practitioners (compared to practitioners working for public institutions) tended to garner more positive attitudes toward adopting EBPs (Aarons et al., 2012) and professionals with higher caseloads associated EBP with more burden (Aarons et al., 2012; Okamura et al., 2018). Older practitioners considered EBP less relevant for job security and observed less organizational support for learning new EBPs (Aarons et al., 2009; Okamura et al., 2018; Rye et al., 2019). More experienced practitioners (as compared to less experienced) perceived therapy more often as a balance between art and science (Aarons et al., 2012), were less open to trying new interventions, and were less willing to use more structured or manualized interventions (Aarons et al., 2010).

Most research on attitudes and the link between attitudes and usage of EBPs has been conducted in HIC. Whether there are differences in the attitudes between professionals in LMIC and HIC is mostly unknown. Recently one study has been conducted in Bangladesh a LMIC (Pervin & Hagmayer, 2022). It found that many Bangladeshi professionals who are working with children and adolescents had positive attitudes towards EBP and the usage of EBPs for children with ASD. Unfortunately, the findings of this study could not be directly compared to findings on attitudes from HIC as measurement invariance of instruments had not been established or different instruments had been used for assessments. Thus, we do not know, whether differences between HIC and LMIC were affected by methodological differences.

The aim of the present study was to investigate potential differences in attitudes towards EBPs, in the number and types of EBPs used, and in the relations between demographic factors, attitudes, and EBPs between mental health professionals providing treatment to autistic children and adolescents in Bangladesh and Germany. The same instrument was used and measurement invariance was established. The following research questions were addressed:

1. Do mental health professionals’ attitudes toward EBP, the number and types of EBPs used differ between Germany as a HIC and Bangladesh as a LMIC?
2. Which socio-demographic factors predict professionals’ attitudes in Bangladesh and Germany?
3. Do attitudes predict the number of different types of EBPs used by professionals in Bangladesh and Germany?

Given the many differences between Germany and Bangladesh with respect to cultural activities, language, funding of healthcare, treatment facilities, training facilities, and knowledge as well as awareness about ASD among healthcare providers, we expected to find differences. However, as there was no comparative research before, we were not able to derive specific hypotheses. In consequence, the present research is exploratory in nature.

## Method

### Sample

The current study was part of a larger research project investigating the attitudes of professionals working with autistic children and adolescents and their relation to the usage of EBPs. For the present study, only data from professionals working in a clinical setting providing treatments were included. As these professional engage in active therapeutic interventions, we considered them mental health professionals. We decided to use purposeful sampling and to search directly for professionals who are likely to work with autistic children and adolescents.

#### Sampling Bangladesh

A list of potentially eligible professionals was collected from the websites of the Bangladesh Association of Psychiatrists, Bangladesh Society for Child Neurology, Bangladesh Clinical Psychology Society, Bangladesh School Psychology Society, Bangladesh Psychological Association, Disability service and support centers, Institute of Special Education and Special schools. Inclusion criteria were the following: participants had to be (i) licensed mental health professionals (psychiatrists, pediatric neurologists, clinical psychologists, psychotherapists, counselors), and/or special teachers, (ii) they had to work with autistic children and adolescents or supervise respective professionals, (iii) they had to be fluent in Bangla or English, and (iv) they had to consent to participate in the anonymous study. Potentially eligible participants were contacted by email or phone. Of the initially identified 302 professionals, 105 did not meet the inclusion criteria. Of 197 eligible professionals, 157 gave informed consent to participate in the study. The survey was completed by 150 participants (see Pervin & Hagmayer, 2022, for more details). Of these participants, 101 worked in a clinical setting, 44 in a special school, and 5 did not provide respective information. The 101 mental health professionals were included in the current study.

#### Sampling Germany

A list of potentially eligible participants and organizations (i.e., associations of professionals, therapy centers, specialized clinics) was collected from respective websites (Psychotherapie-Informationsdienst, Deutsche Psychoanalytische Vereinigung, Verband Sonderpädagogik, Deutscher Verband für Ergotherapie, Bundesverbandes für Ergotherapeuten in Deutschland, Informationsportal Frühförderstellen.de, Bundesverband autismus Deutschland, Berufsverband für Kinder- und Jugendpsychiatrie, Psychosomatik und Psychotherapie, Bundesarbeitsgemeinschaft der Leitenden Klinikärzte für Kinder- und Jugendpsychiatrie, Psychosomatik und Psychotherapie, Bundespsychotherapeutenkammer, Psychotherapeutenkammer Niedersachsen, Kassenärztliche Vereinigung Niedersachsen, Ärztekammer Niedersachsen, see Hansmann, 2022, for further details). 1127 professionals and organizations were contacted by email and asked to participate. In addition, 159 practices were contacted by mail. Inclusion criteria were the same as for the Bangladeshi sample. Of the 255 respondents, who provided informed consent, 10 did not meet the inclusions criteria and 40 did not complete the survey. These respondents were excluded. Of the remaining 205 participants, 14 worked in a special school setting and were excluded for the purpose of the current study leaving 191 participants.

### Measures

#### Demographic Characteristics

Participants’ gender, age, years of experience (the number of years working as a mental health professional), caseload per year (the number of ASD patients worked with on average per year), workplace, professional background, and theoretical orientation were collected.

#### Attitudes

The Evidence-based Practice Attitude scale (EBPAS-36, Rye et al., 2017) was used that measures mental health and social service providers’ attitudes towards adopting EBPs. It was validated in both US and Norwegian samples (Aarons et al., 2012; Rye et al., 2017). Rye et al. (2017) confirmed the factor structure of the EBPAS-36 which evidenced good internal consistency via Cronbach’s alphas for the US and Norwegian samples. In the US sample, Cronbach’s alphas ranged between 0.60 for the divergence subscale and 0.91 for the requirements subscale. The remaining subscales had Cronbach’s alphas above 0.70 (0.81, 0.75, 0.90, 0.77, 0.74, 0.76 and 0.82 respectively). Internal consistency for the Norwegian sample EBPAS-36 ranged from 0.61 for the Appeal subscale to 0.92 for the requirements subscale. The Divergence, Fit and Balance subscales had lower Cronbach’s alphas values (0.68, 0.62 and 0.64 accordingly) and the remaining subscales alphas were above 0.70 (Openness = 0.76, Limitations = 0.85, Burden = 0.74, Job security = 0.86). The EBPAS-36 had not been validated in the context of Bangladesh and Germany prior to the collection of data. Recently, a translation and validation study of a German version of the EBPAS-36 has been published showing good psychometric properties. The internal consistency for the subscales (0.65–0.89), indicating high reliability (Szota et al., 2021). The EBPAS-36 has 12 subscales, with 3 items each. For the present survey, nine subscales (openness, appeal, divergence, limitations, fit, balance, burden, requirements, and job security) were selected as being appropriate for the context of Germany and Bangladesh. Participants respond to respective statements using a five-point Likert scale ranging from 0- “not at all” to 4- “to a very great extent”.

The English version of EBPAS-36 was translated into Bangla by the first author (MP) and into German by the second author (NMH), and back-translated by the second and third author, respectively. The process included several rounds of translation and back-translation with rigorous comparisons between the original and the translated version according to guidelines for cross-cultural translation, adaptation, and validation of instruments (Sousa & Rojjanasrirat, 2011; WHO, 2019). For the Bangla version, a pre-test with mental health clinicians working at the University of Dhaka and mental health clinicians working at the University of Goettingen for the German version was conducted to ensure that all items were understood correctly. The questionnaire was pre-tested by five participants per country.

#### Perceived Barriers

Thirteen new items were developed to assess perceived barriers to the usage of EBPs. The items were based on a recent review of potentially relevant factors (Lau et al., 2015). Responses to these items were not considered in the present study.

#### Current Use of EBPs

In this section of the survey, participants were presented with nine EBPs for ASD. These treatments were established as evidence-based by the National Standards Project (NSP) and the National Professional Development Center (NPDC) (Wong et al., 2015) and a systematic review of medical treatments for children with ASD (McPheeters et al., 2011). The nine types of treatments were: behavioral interventions, cognitive behavioral interventions, comprehensive behavioral treatments for young children, language training, naturalistic teaching strategies, parent training, peer training, social skills training, and antipsychotic medications. Examples were given in the survey to describe each type of treatment. Participants were asked to tick a box when they currently used the type of treatment.

### Procedure

Data were collected by a paper survey administered by research assistants in Bangladesh and an online as well as a paper survey in Germany. We decided to use these different methodologies to ensure a sufficient sample size in Bangladesh, where the total number of potential participants is rather low. By contrast, the number of potential participants in Germany is very large, as explained above. Therefore, we decided to use a widely distributed online survey in combination with a smaller postal survey.

In Bangladesh, potential participants were contacted by phone or email and informed about the study. When professionals agreed to participate, they were visited by a research assistant who obtained informed consent before providing the printed questionnaire. Questionnaires were available in Bangla and English, but all participants preferred the Bangla version. Participants filled in the questionnaire on their own. Research assistants provided clarifications when questions arose and thanked participants for completing the survey. All raw data were stored securely on a server of the University of Dhaka.

In Germany, potential participants were contacted by email and informed about the study. A link to an anonymous online survey was provided. The online survey first informed participants about the study, their rights as participants, and data protection. Participants were asked to provide their informed consent by pressing a respective button. The online survey was constructed using LimeSurvey (2021) and hosted on a server of the University of Goettingen. Paper surveys were sent out to professionals working in private practices. The contacted practitioners received a cover letter, an information sheet on the study, a printed paper version of the questionnaire, and a prepaid envelope to return the questionnaire. The explanation of the study’s purpose and the participants’ rights in the paper version corresponded to the online version. Data were collected anonymously. All raw data were stored securely at the University of Goettingen, Germany.

### Ethics

The present study was approved by the Ethics Committee of Bangladesh Medical Research Council Dhaka, Bangladesh (BMRC; Ref: BMRC/NREC/2019-2022/386). The Internal Review Board of the Georg Elias Mueller Institute for Psychology at the University of Goettingen adopted the approval by the BMRC. As explained in the previous section, all participants gave their informed consent. All data were collected anonymously.

### Statistical Analyses

Statistical analyses were carried out using R (R Core Team, 2020). *P*-values less than 0.05 were considered statistically significant. Scores of the subscales of the EBPAS-36 were computed as described in the handbook (Rye et al., 2017). Before comparisons could be made between Bangladesh and Germany, it was crucial to establish measurement invariance (MI) for the subscales of the EBPAS. First the internal consistencies were assessed by Cronbach’s alpha for each country. Next, confirmatory factor analyses (CFA) were computed for each country individually to test the assumed latent factor structure of the nine EBPAS-36 subscales used in the present survey. Finally, multi-group confirmatory factor analyses were computed to test for configural, metric, and scalar measurement invariance (MI). Scalar MI is required before means can be compared meaningfully (Putnick & Bornstein, 2016).

It turned about that MI could not be established for all nine subscales of the EBPAS. Therefore, a second set of analyses was computed for the four subscales of the EBPAS which had a good internal consistency in both countries. Again, confirmatory factor analyses for each country and a multi-group confirmatory factor analysis across countries were used to investigate MI. If necessary, assumptions were relaxed to establish at least partial scalar MI.

To answer Research Question 1a concerning differences in attitudes between countries, the means of the four EBPAS subscales for which partial scalar MI was given were compared using t-Tests. The significance level was adjusted to 0.0125 to correct for multiple testing. Research Question 1b concerned the number of EBPs used in Bangladesh and Germany. Mean numbers of different EBPs were compared by t-Test. Research Questions 1c concerned differences in the usage of different types of EBPs. Differences between frequencies of usage for the nine individual EBPs were tested by Fisher’s exact test. The significance level was adjusted to 0.0056 to correct for multiple testing.

To answer Research Question 2, which inquired about socio-demographic predictors of attitudes, we computed multi-level models for each of the four EBPAS subscales for which partial scalar MI could be established. Predictors were age, gender, caseload per year (as a categorical variable), professional background (psychiatry and psychology vs. other), and theoretical background (cognitive behavioural or behavioural vs. eclectic vs. other). We excluded the years of experience as a predictor due to a high correlation (> 0.8) with age. Country was used as a grouping variable, as participants were nested within country. Model comparisons (analyses of deviance) were used to find out which predictors were significant. Finally, model comparisons (fixed vs. random slope models) were used to find out whether the significant predictors differed in their predictiveness across countries.

Research Question 3 asked whether attitudes predict the number of different types of EBPs used in Bangladesh and Germany. Again, we used multi-level modelling with country being the grouping variable. The four attitude subscales for which partial scalar MI could be established were used as predictors. As before, model comparisons were used to identify which predictors were significant and whether their predictiveness differed between countries.

## Results

### Participants

A total of 292 professionals working in a clinical setting and providing treatments to children and adolescents with ASD were included in the current study (*n* = 191 for Germany; *n* = 101 for Bangladesh). Table 1 provides an overview of the socio-demographic data. Participants from Bangladesh were on average 32.7 years old, participants from Germany 44.5 years. The mean years of experience were 5.9 years in Bangladesh and 16.5 years in Germany. In both countries the majority of participants were female. As the number of cases per year varied widely, in Bangladesh numbers were considerably higher with more than a third of participants seeing more than 100 patients with ASD per year. In Germany a vast majority of participants worked with up to 10 patients and very few with more than 100 patients. In Germany almost half of the participants had a degree in psychiatry, medicine, or psychology, while only one in four did in Bangladesh. The theoretical orientation of most German participants was cognitive-behavioral (79.8%), followed by humanistic (32.5%), psychoanalytic or psychodynamic (18.8%), while, the Bangladeshi respondents’ theoretical orientations were eclectic (49.5%) and cognitive-behavioral (37.6%). In both countries, most participants worked in an outpatient setting. In Germany, slightly more than a third of participants (37.7%) worked in their own practice, while only 15.8% of the participants from Bangladesh did.

**Table 1:** Socio-demographic characteristics of the participants

Characteristics	Bangladesh		Germany	
	Mean	SD	Mean	SD
Age	32.7	8.2	44.5	11.3
Years of experience	5.9	10.2	16.5	10.2

	N	Percent	N	Percent
Gender				
Female	75	74.3	134	70.2
Male	26	25.7	52	27.2
Non-binary	–	–	3	1.6
NA	–	–	2	1.0
Caseload (per year)				
1–10	36	35.6	130	68.0
11–100	24	23.8	49	25.7
More than 100	37	36.6	3	1.6
Professional background				
Medicine (incl. psychiatry)	9	8.9	20	10.5
Psychology	17	16.8	74	38.7
Special education	4	4.0	33	17.3
Other or double degree	69	72.3	64	33.5
Theoretical background^a^				
Psychoanalytic/psychodynamic	3	3.0	36	18.8
Cognitive-behavioral	38	37.6	162	79.6
Humanistic	7	6.9	62	32.5
Eclectic	50	49.5	26	13.6
Other	19	18.8	35	18.3
Setting^a^				
Outpatient	81	80.2	173	90.6
Inpatient	16	15.8	34	17.8
NA	4	4.0	–	–
Private practice				
Works in own private practice	16	15.8	72	37.7

^a^Multiple options could be chosen. Therefore, numbers add to more than 100%

### Measurement Invariance of the Subscales of the Evidence-Based Practice Attitude Scale

For the Bangladeshi sample, Cronbach’s *α* ranged between 0.23 and 0.91, and for the German sample, consistencies of the nine subscales of the EBPAS-36 ranged from *α* = 0.45 to *α* = 0.92. Four subscales of the EBPAS showed a Cronbach’s *α* >  = 0.70 in both samples: Openness, Appeal, Requirements, and Job security (see Table 2 for details). Therefore, measurement invariance was tested for a model with these four subscales only. It turned out that the fit of the model in the German sample was good, χ^2^ = 98.5***, CFI = 0.96, TLI = 0.94, RMSEA = 0.074, SRMR = 0.058, and excellent in the Bangladeshi sample, χ^2^ = 59.1, CFI = 0.98, TLI = 0.98, RMSEA = 0.048, SRMR = 0.046. The results for the multigroup analyses are shown in Table 3. It turned out that for these four subscales at least partial scalar invariance could be achieved. Scalar invariance (i.e. same structure of latent factors, same factor loadings, and same intercepts) could not be fully obtained. The respective model was significantly worse than the models making less strict requirements, although the model fit was still very good (see Table 3). Releasing some assumptions, partial scalar invariance resulted (see Note of Table 3 for Details). This means that the answers to the two subscales Requirements and Job security can be directly compared between Bangladesh and Germany, while the comparisons for the subscales Appeal and Openness should be interpreted with some caution, but a comparison is possible.

**Table 2:** Internal consistencies of the nine subscales of the EBPAS-36

Sub-scales	Bangladesh	Germany
	Cronbach's *α*	Cronbach's *α*
Openness	0.83	0.77
Divergence	0.23	0.45
Appeal	0.70	0.75
Limitations	0.55	0.52
Fit	0.83	0.63
Balance	0.62	0.52
Burden	0.60	0.72
Job-security	0.85	0.83
Requirements	0.91	0.92

**Table 3:** Analysis of measurement invariance MI by multi-group confirmatory factor analyses

CFA models	χ^2^	df	TLI	CFI	RMSEA (90% CI)	AIC
Configural MI	153.3***	96	0.97	0.95	0.066 [0.046–0.085]	7755.6
Metric MI	169.5***	104	0.96	0.95	0.068 [0.049–0.086]	7755.8
Partial scalar MI^a^	171.5***	107	0.96	0.95	0.066 [0.047–0.084]	7751.8
Scalar MI	210.3***	112	0.94	0.93	0.080[0.063–0.096]	7780.6

Model comparisons	χ^2^	df	*p*			
Configural vs. metric	16.2	8	0.04*			
Configural vs. partial scalar	18.2	11	0.08			
Weak vs. partial scalar	1.95	3	0.58			

Model contains four subscales of the EBPAS: openness, appeal, requirements, and job security

^a^For the partial scalar invariance model the following assumptions were released: subscale appeal: same loading for one item and same intercepts of two items, subscale openness: same intercepts for items (see main text more information on specific items)

*p < 0.05, **p < 0.01, ***p < 0.001

### Research Question 1a: Differences in Attitudes Towards EBP Between Bangladesh and Germany

Mean ratings (and SD) of the EBPAS-36 subscales in Bangladesh and Germany can be found in Table 4. Note that only the four subscales are presented for which partial scalar MI could be established. The t tests revealed that German and Bangladeshi participants differed significantly on three subscales (Appeal, Requirement, and Job security).The findings revealed that German participants had a significantly higher score (*M* = 3.34) than Bangladeshi participants (*M* = 3.08) on the Appeal subscale, indicating that German professionals were more likely than Bangladeshi professionals to adopt a new practice if it were intuitively appealing, made sense to them, could be used correctly, or were used by colleagues who are happy with it. For the Requirements and Job security subscales means were significantly lower for the German participants. This implies that German mental health professionals were less likely to adopt a new practice if it was required by an agency, supervisor, or state, and that they considered learning new EBPs less relevant for job security than Bangladeshi participants. There were no significant differences between the German and Bangladeshi professionals on the Openness subscale.

**Table 4:** Attitudes towards EBPs of mental health professionals in Bangladesh and Germany. Mean ratings (SD and CI) of four subscales of the EBPAS-36

EBPAS-36 scale	Bangladesh			Germany			Comparison		
	*M*	*SD*	CI	*M*	*SD*	CI	t	df	*p*
Openness	2.92	0.82	[2.76–3.08]	3.04	0.74	[2.94–3.15]	− 1.289	287	0.198
Appeal	3.08	0.76	[2.92–3.24]	3.34	0.61	[3.24–3.43]	− 2.934	157.8	0.004**
Requirements	3.10	1.05	[2.89–3.31]	2.49	1.06	[2.33–2.64]	4.642	286	<0.001***
Job security	3.21	0.85	[3.05–3.39]	1.55	1.12	[1.39–1.71]	14.098	250.9	< 0.001***

Partial scalar measurement invariance was achieved, but some assumptions for the subscales Openness and Appeal had to be released. Therefore, the respective comparisons of means should be interpreted with caution

*p < 0.05, ** *p* < 0.01, *** *p* < 0.001

### Research Question 1b: The Number of EBPs Used in Bangladesh and Germany

**Fig. 1:**
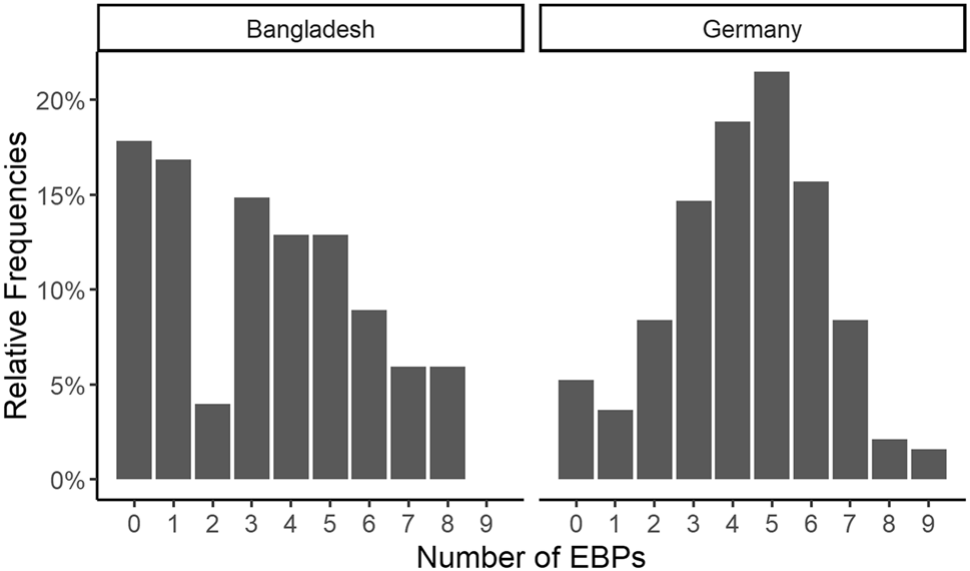
Number of different types of EBPs used in Bangladesh and Germany

As Fig. 1 shows, German participants used more types of EBPs than Bangladeshi participants. Interestingly, almost eighteen percent of Bangladeshi clinicians (n = 18) did not use any of the nine types of interventions, whereas only 5% of German professionals (n = 10) reported that they did not use any of them. The mean numbers differed significantly (Germany: M = 4.31, SD = 1.97, Bangladesh: M = 3.28, SD = 2.49, *t* (167.98) = 3.61, *p* = 0.0004). Thus, professionals seem to use a broader variety of different types of treatment for autistic children and adolescents in Germany than in Bangladesh.

### Research Question 1c: Types of EBPs Used in Bangladesh and Germany

Table 5 shows how many participants in Bangladesh and Germany reported using each individual type of EBP. The use of behavioral interventions was reported by more than 80% of the respondents (n = 161) in Germany compared to 45% in Bangladesh. In addition, more than 75% of the respondents in Germany (79.1%) indicated that they were currently using social skills training, 64.9% were implementing parent training, 62.8% were using cognitive behavioural interventions, and 61% were using language training. These types of interventions were used less often by Bangladeshi respondents. The usage of these types of interventions differed significantly between the two countries (see Table 5 for details). There were no significant differences, however, with respect to comprehensive behavioral interventions, naturalistic teaching strategies, peer training, and antipsychotic medications. Interestingly, comprehensive behavioural interventions and antipsychotic medications were used only by a small number of respondents in both countries.

**Table 5:** EBPs currently used by mental health professionals for children and adolescents with ASD in Bangladesh and Germany

Intervention type	Bangladesh		Germany		Odds ratio and Fisher’s exact test
	n	%	n	%	
Behavioral interventions (e.g., imitation training, reinforcement schedules, video modeling)	45	44.6	161	84.3	6.63***
Cognitive behavioral intervention(i.e., behavior modification and cognitive restructuring)	40	39.6	120	62.8	2.57***
Comprehensive behavioral treatment for Young Children (e.g., LOVAAS program)	18	17.8	22	11.5	0.60
Language training (e.g., Functional verbal communication)	46	45.5	61	31.9	0.56*
Naturalistic teaching strategies (e.g., verbal prompting)	39	38.6	86	45.0	1.30
Parent training (i.e., individual or group training by using training manuals)	47	46.5	124	64.9	2.12*
Peer training (e.g., Project LEAP, peer networks, integrated play groups)	24	23.8	62	32.5	1.54
Social skill training (i.e., behavioral interventions to improve social and communicative competencies)	51	50.5	151	79.1	3.68***
Antipsychotic medications (aripiprazole, risperidone, chlorpromazine, prochlorperazine, clozapine)	21	20.8	36	18.8	0.89

*p < .05, **p < .01, ***p < .001

### Research Question 2: Differences in the Predictive Values of Demographic Characteristics for Attitudes Towards EBP Between Bangladesh and Germany

The results of all analyses are presented in Table 6. Relations between demographic characteristics, which turned out to be significant predictors of the respective subscales, are depicted in Figs. A1–A4 in the Online Appendix.

**Table 6:** Multi-level models for the EBPAS-36 “openness”, “appeal”, “requirements” and “job security” subscales and ANOVA of potential predictors age, gender, caseload per year (categories), professional background (medicine or psychology vs. other), theoretical background (cognitive behavioral vs. eclectic vs. other), work in own practice

Openness					
Models	AIC	Test	Likelihood ratio	*p*	*Pseudo R*^*2*^
1 Random intercept	623.2	1 vs Nullmodel	0.0 1	0.984	< 0.001
2 Random intercept fixed effects	623.2	2 vs. 1	16.0	0.042	0.058
3 Random intercept random slope age	622.8	3 vs 2	4.363	0.113	0.016
4 Random intercept random slope caseload per year	627.3	4 vs. 2	5.922	0.313	0.022

ANOVA for predictors	df	Chi-squared	*p*		
Age	1	7.93	0.005		
Gender^a^	1	1.11	0.291		
Caseload per year	2	6.03	0.049		
Professional background	1	0.23	0.630		
Theoretical background	2	2.78	0.249		
Works in own practice	1	1.56	0.212		

Appeal					
Models	AIC	Test	Likelihood ratio	*p*	*Pseudo R*^*2*^
1 Random intercept	542.2	1 vs Nullmodel	5.522	0.019	0.021
2 Random intercept fixed effects	533.7	2 vs. 1	24.517	0.002	0.088
3 Random intercept random slope gender	537.3	3 vs 2	0.414	0.813	0.002
4 Random intercept random slope caseload per year	529.7	4 vs. 2	14.009	0.016	0.051

ANOVA for predictors	df	Chi-squared	*p*		
Age	1	0.001	0.980		
Gender^a^	1	6.065	0.014		
Caseload per year	2	8.575	0.014		
Professional background	1	1.725	0.189		
Theoretical background	2	2.127	0.345		
Works in own practice	1	3.392	0.066		

Requirements					
Models	AIC	Test	Likelihood ratio	*p*	*Pseudo R*^*2*^
1 Random intercept	785.4	1 vs Nullmodel	14.894	< 0.001	0.055
2 Random intercept fixed effects	765.9	2 vs. 1	35.451	< 0.001	0.125
3 Random intercept random slope own practice	767.2	3 vs 2	2.673	0.263	0.010
4 Random intercept random slope caseload per year	770.4	4 vs. 2	5.531	0.355	0.021

ANOVA for predictors	df	Chi-squared	*p*		
Age	1	1.360	0.244		
Gender^a^	1	2.414	0.120		
Caseload per year	2	22.887	< 0.001		
Professional background	1	0.000	0.993		
Theoretical background	2	1.887	0.389		
Works in own practice	1	9.813	0.002		

Job security					
Models	AIC	Test	Likelihood ratio	*p*	*Pseudo R*^*2*^
1 Random intercept	763.2	1 vs Nullmodel	126.600	< 0.001	0.383
2 Random intercept fixed effects	771.6	2 vs. 1	7.568	0.477	0.028
3 Random intercept random slope age	775.6	3 vs 2	0.037	0.982	< 0.001

ANOVA for predictors	df	Chi-squared	*p*		
Age	1	3.876	0.049		
Gender^a^	1	1.474	0.225		
Caseload per year	2	0.650	0.722		
Professional background	1	0.098	0.754		
Theoretical background	2	0.561	0.755		
Works in own practice	1	0.001	0.978		

#### EBPAS-36 Openness

The comparison of a Nullmodel and a model with a random intercept model for country showed no significant improvement in model fit, χ^2^ (1) = 0.001, *p* = 0.984. Adding demographic characteristics as predictors with fixed effects (age, gender, caseload, professional background, theoretical background, work in own practice) improved model fit, χ^2^ (8) = 16.037, *p* = 0.042. An analysis of deviance investigating the individual predictors showed age, χ^2^ (1) = 7.935, *p* = 0.005, and caseload, χ^2^ (2) = 6.033, *p* = 0.049, to be significant predictors of openness towards EBP. Adding random slopes for age, χ^2^ (2) = 4.363, *p* = 0.113, as well as caseload, χ^2^ (5) = 5.922, *p* = 0.313, did not significantly improve model fit. The relations between age and openness as well as caseload and openness are visualized in the Fig. A1 in the Online Appendix. The figure shows that overall age was negatively related to openness, but the relation tended to be positive in Bangladesh and negative in Germany (due to the larger number of German participants and their higher age, the overall relation was negative). For caseload, openness tended to increase with caseload in Bangladesh, while openness tended to decrease in Germany when more than 100 youth were seen.

#### EBPAS-36 Appeal

Comparing a Nullmodel to a model with a random intercept for country showed a significant improvement in model fit, χ^2^ (1) = 5.522, *p* = 0.019. This reflects the higher ratings of appeal in Germany (see Table 4). Adding demographic characteristics as predictors with fixed effects to the random intercept model significantly improved model fit, χ^2^ (8) = 24.517, *p* = 0.002. An analysis of deviance showed gender, χ^2^ (1) = 6.065, *p* = 0.014, and caseload, χ^2^ (2) = 8.575, *p* = 0.014, to be significant predictors of the appeal of EBP. A random slope for gender, χ^2^ (2) = 0.414, *p* = 0.813, did not significantly improve model fit, which indicates that there were no differences between countries. By contrast, a random slope for caseload, χ^2^ (5) = 14.009, *p* = 0.016, did improve model fit. Fig. A2 visualizes the relations. Interestingly, appeal increased with caseload in Bangladesh, while there was a tendency towards a negative relation in Germany.

#### EBPAS-36 Requirements

The comparison of a Nullmodel with model with a random intercept for country showed significant improvement in model fit, χ^2^ (1) = 14.894, *p* < 0.001. As shown in Table 4, ratings for requirements were significantly higher in Bangladesh. Adding demographic characteristics as predictors with fixed effects to the random intercept model resulted in further significant improvement in model fit, χ^2^ (8) = 35.451, *p* < 0.001. An analysis of deviance found that working in one’s own practice, χ^2^ (1) = 9.813, *p* = 0.002, and caseload, χ^2^ (2) = 22.887, *p* < 0.001, were significant predictors for attitudes regarding requirements. Neither a random slope for working in one’s own practice, χ^2^ (2) = 2.673, *p* = 0.263, nor for caseload, χ^2^ (5) = 5.531, *p* = 0.355, showed significant improvement in model fit. The relations are shown in Fig. A3 in the Online Appendix. While in Bangladesh the willingness to use EBPs when required clearly increased with caseload, no such relation was visible in Germany. In both countries the willingness to use EBPs when required was lower for professionals working in their own practice.

#### EBPAS-36 Job Security

The comparison of a Nullmodel with model with a random intercept for country showed significant improvement in model fit, χ^2^ (1) = 126.600, *p* < 0.001. Table 4 shows that mean ratings were substantially higher in Bangladesh. Adding demographic characteristics as predictors with fixed effects to the random intercept model, did not significantly improve model fit, χ^2^ (8) = 7.568, *p* = 0.477. Thus, demographic variables overall did not predict the willingness to learn new EBPs to improve job security. An analysis of deviance, however, found that age, χ^2^ (2) = 3.876, *p* = 0.049, was a significant predictor for attitudes regarding job security. A random slope for age, χ^2^ (2) = 0.037, *p* = 0.982, did not improve model fit. As Fig. A4 in the Online Appendix shows there was slight decrease in attitudes concerning job security, which was mainly driven by the German participants who were older than the Bangladeshi participants.

### Research Question 3: Predictive Values of Attitudes for the Number of Different Types of EBPs Used by Professionals in Bangladesh and Germany

The comparison of a Nullmodel with a model with a random intercept for country showed a significant improvement in model fit, χ^2^ (1) = 7.104, *p* = 0.008. This finding reflects that German participants used a broader variety of evidence-based practices (see Fig. 1). Adding the scores of the EBPAS-36 subscales Openness, Appeal, Requirements, and Job Security as predictors with fixed effects to the random intercept model significantly improved model fit, χ^2^ (8) = 98.232, *p* < 0.001. An analysis of deviance showed that appeal was the only significant predictor for the number of different EBPs used by clinicians, χ^2^ (1) = 8.087, *p* = 0.004 (see Table 7). A random slope for Appeal, χ^2^ (2) = 2.561, *p* = 0.278, did not significantly improve model fit. Figure A5 in the Online Appendix depicts the relation between appeal and the number of EBPs in Bangladesh and Germany. In both countries the relation was positive.

**Table 7:** Multi-level models for the number of EBPs used and ANOVA of potential predictors EBPAS-36 four subscales “openness”, “appeal”, “requirements” and “job security”

Number of EBPs					
Models	AIC	Test	Likelihood ratio	*p*	*Pseudo R*^*2*^
1 Random intercept	1187.8	1 vs nullmodel	7.104	.008	.026
2 Random intercept fixed effects	1097.6	2 vs. 1	98.232	< .001	.321
3 Random intercept random slope appeal	1099.0	3 vs 2	2.561	.278	.010
ANOVA for predictors	df	Chi-squared	*p*		
Openness	1	0.186	.666		
Appeal	1	8.087	.004		
Requirements	1	1.939	.164		
Job security	1	3.610	.057		

## Discussion

The aim of this study was to investigate potential differences in professionals’ attitudes towards EBP, in the number and types of EBPs used, and in the relations among demographic characteristics, attitudes, and EBPs between mental health professionals proving treatment to autistic children and adolescents in Bangladesh and Germany. While Bangladesh is a LMIC with only few mental health professionals and a rather underdeveloped health care system especially with respect to mental health, Germany is a HIC with many mental health professionals and institutions specialized to delivering care and treatment to children and adolescents with developmental disorders including ASD. Therefore, it is interesting to know, whether there are differences in attitudes towards EBP, the usage of different EBPs, and the relations among demographic characteristics, attitudes, and the number of different EBPs used.

To allow for a meaningful comparison, we focused on mental health professionals working in a clinical setting delivering care to autistic children and adolescents. We chose such a specific group of participants to ensure that potential differences were not due to the work of participants or the treatments and practices which are evidence-based in their respective field. In principle, the evidence is the same for Bangladesh and Germany, although very little of the evidence comes from LMIC (see Pervin et al., 2022).

Second, we tested measurement invariance for the subscales of the structured questionnaire we used to assess participants’ attitudes before making comparisons. The EBPAS-36 (Rye et al., 2017) is well established and evaluated in Western HIC. The present study is the first to investigate psychometric properties and internal consistency of nine subscales of EBPAS-36 in Bangladesh and Germany. The internal consistencies of the subscales ranged from acceptable to good, with the subscales Divergence, Limitations, and Balance demonstrating the lowest internal consistencies in the German sample. The subscales Divergence and Limitations had low consistency in the Bangladeshi sample, which mirrors findings in a US sample (Rye et al., 2017). The EBPAS-36 had adequate psychometric properties in a US (clinics providing mental health services) and a Norwegian sample (psychologists, psychiatric nurses, and psychology students; Rye et al., 2017). In Germany, one study reported good item properties, internal consistencies, and convergent validity (Szota et al., 2021). This study, however, used a different translation. Testing for measurement invariance, we found no invariance across countries for all nine subscales. This entails that the constructs measured had a different meaning or structure in the two countries and/or the questions were understood differently (Putnick & Bornstein, 2016). For four sub-scales, partial scalar invariance could be established: Openness, Appeal, Requirements, and Job security*.* Therefore, only these four subscales were used for comparisons between countries and other statistical analyses.

Our first research question (RQ 1a) concerned potential differences in attitudes towards EBP. Overall, the findings indicate a rather positive attitude of professionals in Germany and Bangladesh. A comparison of the scores of the four subscales showed that practitioners in both countries were similarly open to using EBPs. Other studies from LMIC and upper middle-income countries (UMIC) (Busse et al., 2021; Chetwin, 2018; Hall et al., 2022; Vasudevan et al., 2020) reported higher levels of openness than studies from HIC (Aarons et al., 2010; James et al., 2019; Rye et al., 2017; Szota et al., 2021). It is important to note, however, that none of these studies made direct comparisons establishing measurement invariance before.

With respect to appeal, German professionals gave significantly higher ratings than Bangladeshi professionals, i.e. they said they would be more likely to use these EBPs if they seemed personally useful, if they felt sufficiently trained, and if colleagues were satisfied with them. One possible explanation might be that certified mental health professionals in Germany are rather free in using new treatments when there is evidence for them. This is especially true for those working in their own practice. Bangladeshi professionals, by contrast, often work in institutions with many regulations, which entails that they might be less flexible to try new things even when they are appealing. This finding is consistent with previous findings from Germany that attitudes of social workers towards EBP were mostly positive. They would use new methods if they made sense, were appealing, and practitioners received enough training to use the methods correctly (James et al., 2019).

Concerning requirements, German professionals showed a significantly lower willingness to adopt an EBP if it was required at work, by their supervisor, or the state than Bangladeshi professionals. Other studies from HIC (Aarons et al., 2010; James et al., 2019; Rye et al., 2017; Szota et al., 2021) and from UMIC as well as LMIC (Busse et al., 2021; Chetwin, 2018; Hall et al., 2022; Vasudevan et al., 2020) also showed more positive attitudes on the requirement subscales in the latter countries, with the exception of Indonesia, but no direct comparisons exist in the literature. One possible explanation for the findings is that certified mental health clinicians in Germany are rarely required to use a specific type of treatment. In fact, there are no mandatory guidelines. Not being used to requirements, they may be less willing to concede and adopt them. By contrast, Bangladeshi professionals may welcome new requirements, which provide guidance and could simplify their daily work.

German professionals assigned a lower value to job security than Bangladeshi professionals. This means they were less willing to learn a new EBP to keep or find a new job. One explanation might be that a majority of German participants had a cognitive behavioral theoretical background and CBT practices are generally evidence-based. Thus, they may have felt little necessity to learn new EBPs. In addition, many had long years of experience and a third worked in their own practice, which entails that they had a high level of job security. By contrast, many Bangladeshi professionals claimed various theoretical backgrounds. They were also younger and had less experience. Therefore, the learning of new EBPs may be important for their future career.

Our first research question (RQ1b & RQ1c) also asked about differences in the number and the types of EBPs used in the two countries. We found that German professionals reported the use of a higher number of different EBPs than Bangladeshi professionals. Most of the EBPs (e.g. social skill training, cognitive behavioral interventions, behavioral interventions, and parent-mediated interventions as well) were more likely to be used by mental health professionals in Germany than in Bangladesh. There are numerous possible explanations for this finding. One is differences in training. More participants in Germany had a CBT background. Another possible explanation is that professionals in Bangladesh might be reluctant to use certain EBPs as most evidence comes from HIC (NPDC 2017a, 2017b; Parsons et al., 2017; Pervin et al., 2022; Ratliff-Blake & Therrien, 2021; Reichow et al., 2013; Steinbrenner et al., 2020). A final reason may be related to financial constraints. For example, comprehensive treatment programs, which are highly costly, were used infrequently in both settings. An interesting case are parent trainings. Although half of the participants in Bangladesh reported using them, more did in Germany. This is surprising, as parent-mediated interventions are commonly used LMIC due to their cost efficiency (Blake et al., 2017). In a previous study from the same project, we found that a broader variety of EBPs was used in special school settings than in clinical settings in Bangladesh (Pervin & Hagmayer, 2022). This was also true for parent trainings. The infrequent usage of antipsychotic medications (e.g., Aripiprazole, Risperidone) in both countries might be due to the low number of psychiatrists and other physicians participating. Non-medical professionals are not allowed to use medications in either country. In addition, professionals may be reluctant to use them due to their significant side effects (Ching & Pringsheim, 2012; Hirsch & Pringsheim, 2016; Pervin et al., 2022).

Research Question 2 inquired about the relation between demographic characteristics and attitudes as well as potential differences in these relations between countries. It was analysed whether age, gender, caseload, work in one’s own practice, and professional as well as theoretical background predicted attitudes. We found that demographic characteristics were associated with the intuitive appeal of EBPs and the willingness to implement them when required. The relation was marginally significant for openness.

Caseload was the most important predictor being related to appeal, openness, and requirements. Overall higher caseloads were related to more positive attitudes. The relation, however, seems to vary between countries. Bangladeshi practitioners with higher caseloads reported a higher appeal of EBPs, more openness, and more willingness to follow requirements, while in Germany the relations tended to be negative or non-existent. This difference between countries was significant for appeal. The same, although non-significant difference emerged for openness and requirements. Previous literature from HICs reported that caring for a higher number of patients was associated with more negative attitudes towards EBP (Aarons, 2004; Aarons et al., 2012; Rye et al., 2019). In South Africa, a UMIC, in which practitioners are confronted with a high number of patients, higher caseloads were also associated with more negative attitudes, especially with respect to openness (Magidson et al., 2018). A possible explanation for the positive effect of caseload on Bangladeshi practitioners’ attitudes might stem from the conditions mental health professionals, especially governmental health professionals face. Many are confronted with an inadequate infrastructure and supplies, high caseloads, administrative problems, and patients commonly seeking the help of unqualified private practitioners (Cockcroft et al., 2011; Hall et al., 2022). EBPs, which are derived from clinical research and provide an effective and structured approach to treatment, may be therefore perceived as very attractive, in particular for those that have to provide services to many patients.

Research Question 3 asked about the relation between attitudes and the number of different EBPs used and potential differences in this relation between countries. We found that only the intuitive appeal of EBPs was significantly and positively related to the number of different EBPs used. Professionals, who said that they would use a new EBP when they felt adequately trained, thought that it made sense, and knew that colleagues were happy with it, used a greater variety of EBPs. This was true for both Bangladesh and Germany. This finding is consistent with previous findings that a lack of appealing features hinders the implementation and usage of EBPs (Aarons et al., 2009; Jensen-Doss et al., 2009; Reding et al., 2014).

### Limitations

Some limitations of the present work should be considered. One concern was the low response rate in Germany, which is a familiar problem in online surveys (Van Horn et al., 2009). In addition, contacted professionals may have based their decision to participate depending on their interest in EBPs. Therefore, the composition of the sample may not be representative for mental health professionals working with autistic children and adolescents in Germany. The same problem may be present in Bangladesh, but to a much smaller degree as a much larger proportion of the potentially eligible participants responded to the survey. Nevertheless, we cannot assume that the results are representative (Nübling et al., 2014).

A related concern may be the limited sample size, which did not allow us to make comparisons between different groups of professionals in the two countries (e.g., psychologists vs. psychiatrists). For the present study we focused on participants that worked in a clinical setting in order to make the samples more comparable. In consequence, the overall sample size was slightly below 300. Note that this sample size was sufficient to detect small to medium effects between countries with high statistical power.

Another limitation is that we used different methodologies to collect our data in Bangladesh and Germany. We opted for paper surveys being personally delivered to participants in Bangladesh, and a mixture of a large online survey and a smaller postal survey in Germany. This methodological difference may have affected results.

A limitation was also that we could not establish measurement invariance across countries for all subscales of the EBPAS-36. This might be due to the fact that we had to translate the English version of the questionnaire into local languages as no translations were available when the surveys were conducted. The translations may have resulted in unintended differences in the meanings of particular items. In consequence, we refrained from computing an overall score for the attitude towards EBP. For the four subscales Openness, Appeal, Requirements and Job security subscales we were able to establish partial scalar measurement invariance, which allowed us to use the respective scores in our analyses. The fit of the respective model was very good. Nevertheless, we had to ease a few assumptions for the Openness and Appeal subscale.

### Implications for Implementation of EBPs and Future Research

Despite the limitations outlined in the previous section, this study has implications for the implementation of EBPs and for further research. The differences between the countries indicate that findings in one country cannot be transferred to another country. Therefore, attention should be paid to country-specific factors when formulating implementation strategies for EBPs. Relevant differences were found for the appeal of EBPs and requirements concerning the use of EBPs. The high willingness of Bangladeshi professionals to adopt an EBP when required points out that mandatory guidelines could be successful in Bangladesh. In Germany, by contrast, it might better to avoid requirements and to spend more effort to make EBPs appealing to practitioners. The appeal of EBPs could be increased by providing respective trainings, which may also help to prevent misunderstanding of the concept of evidence-based practice (cf. Evidence-Based Medicine Working Group, 1992; Spies, 2019; Szota et al., 2021). Positive feedback by colleagues using EBPs may also be helpful to increase the appeal. Future studies will have to show, which of these strategies work the best in different countries.

Our study also provides some information for future international comparison studies on professionals’ attitudes. Structured assessment tools for attitudes towards EBPs like the EBPAS-36, which have been successfully translated and validated in various Western HIC are a good starting point. But translating them into the language and the cultural context of non-Western LMIC (or UMIC) in a way that ensures measurement invariance will require new efforts. Respective projects will require substantial funding and the collaboration of practitioners, language experts, and researchers from the respective countries. In addition, heterogeneous samples should be avoided as they may affect measurement invariance negatively. Thus, larger samples from the different countries should be investigated, which would allow researchers to focus on specific sub-groups of practitioners or to control for more potentially relevant factors than we did.

With respect to potentially relevant demographic variables, future studies may want to re-investigate the effect of age and caseload. In addition, the working conditions of the professionals should be investigated in more detail, including working hours, responsibilities, resources, leadership by superiors, on the job training, and the freedom to make decisions (cf. Lau et al., 2015). The findings may indicate effective leverage points for implementation strategies.

## Conclusion

This is the first study that directly compared the attitudes towards EBPs and the usage of different EBPs in a HIC (Germany) and a LMIC (Bangladesh). It also represents one of the first endeavours to explore factors that may influence professionals’ attitudes towards EBP in both countries. Future comparative research needs to examine potential factors within and beyond professionals’ attitudes to generate a more comprehensive understanding of the critical issue of professionals’ attitudes by applying a mixed-methods design. This may help generate strategies to support the more widespread implementation of EBPs, which improve the life of autistic children and adolescents.

## Supplementary Information

Below is the link to the electronic supplementary material.

Supplementary file1 (DOCX 172 kb)

Supplementary file2 (DOCX 25 kb)

## Footnotes

1 The terms evidence-based practice (EBP) and evidence-based practices (EBPs) are often used synonymously in the literature. There is an important distinction, though. EBP was defined as “the integration of best research evidence with clinical expertise in the context of patient characteristics, culture, and preferences.” (American Psychological Association, 2005, p. 5). Therefore, EBP “requires the health professional to consider characteristics of the practice context in which they work” (Hoffman et al., 2017, p. 337). By contrast, EBPs are treatments that have been proven efficacious in research.
