# Supplementary material for: Attitudes Toward and Usage of Evidence-Based Mental Health Practices for Autistic Youth in Bangladesh and Germany: A Cross-Cultural Comparison
Source: J Autism Dev Disord. 2024 Jan 26;55(2):635–51. doi: 10.1007/s10803-023-06223-z (PMC11813821; doi:10.1007/s10803-023-06223-z)
Supplement: Supplementary file 1 — Supplementary file1 (DOCX 172 kb) [file 10803_2023_6223_MOESM1_ESM.docx]

**Evidence-Based Practice Attitude Scale-36 (EBPAS-36; Nine Subscales)**

| **English version** | **Bangla version** | **German version** |
| --- | --- | --- |
| ***Openness*** | | |
| I like to use new types of therapy/interventions to help my clients. | আমি আমার ক্লায়েন্টদের সাহায্য করার জন্য নতুন ধরনের থেরাপি / হস্তক্ষেপ ব্যবহার করতে চাই | Ich nutze gerne neue Behandlungsverfahren um meinen Patienten zu helfen. |
| I am willing to try new types of therapy/interventions even if I have to follow a treatment manual. | আমার যদি চিকিৎসা ম্যানুয়াল অনুসরণ করার সুযোগ থাকে তবে নতুন ধরনের থেরাপি / হস্তক্ষেপ ব্যবহার করতে ইচ্ছুক | Ich bin bereit, neue Behandlungsverfahren auszuprobieren, auch wenn ich ein Manual verfolgen muss. |
| I am willing to use new and different types of therapy/interventions developed by researchers. | আমি গবেষকরা তৈরী করেছেন এমন নতুন ও বিভিন্ন ধরনের থেরাপি / হস্তক্ষেপগুলি ব্যবহার করতে ইচ্ছুক | Ich bin bereit, neue und andere Behandlungsverfahren auszuprobieren, die Forscher  entwickelt haben. |
| ***Divergence*** | | |
| Research-based treatments/interventions are not clinically useful | গবেষণা ভিত্তিক চিকিৎসা / হস্তক্ষেপগুলো চিকিৎসাগতভাবে ব্যবহৃত নয় | Auf Forschung basierende Behandlungsverfahren sind in der klinischen Praxis nicht  nützlich |
| Clinical experience is more important than using manualized therapy/treatment | ম্যানুয়াল থেরাপি / চিকিৎসা ব্যবহার করার চেয়ে চিকিৎসার অভিজ্ঞতা বেশী গুরুত্বপূর্ণ | Erfahrung ist wichtiger als der Gebrauch von manualisierten Behandlungsverfahren |
| I would not use manualized therapy/interventions | আমি ম্যানুয়ালাইজড থেরাপি / হস্তক্ষেপ ব্যবহার করব না | Ich würde keine manualisierten Behandlungsverfahren nutzen |
| ***Balance*** | | |
| A positive outcome in therapy is an art more than a science | থেরাপির ইতিবাচক দিক হচ্ছে এটি একটি শিল্প যা বিজ্ঞান থেকে উন্নত | Ein positives Ergebnis in der Behandlung ist eher Kunst als Wissenschaft |
| Therapy is both an art and a science | থেরাপি একই সাথে একটি শিল্প এবং একটি বিজ্ঞান | Eine Behandlung ist sowohl Kunst als auch Wissenschaft |
| My overall competence as a therapist is more important than a particular approach | একজন চিকিৎসক হিসাবে আমার সামগ্রিক দক্ষতা একটি নির্দিষ্ট পদ্ধতির চেয়ে অধিকতর গুরুত্বপূর্ণ | Meine Kompetenz ist wichtiger als eine spezifische Behandlungsmethode |
| ***Burden*** | | |
| I don’t have time to learn anything new | আমার নতুন কিছু শেখার সময় নেই | Ich habe nicht die Zeit, Neues zu lernen |
| I can’t meet my other obligations | আমি আমার অন্যান্য বাধ্যবাধকতা পূরণ করতে পারছি না | Ich kann meinen anderen Verpflichtungen nicht nachkommen, wenn ich diese nutze |
| I don’t know how to fit evidence-based practice into my administrative work | আমি জানি না কিভাবে আমার প্রশাসনিক কাজের সাথে প্রমাণ-ভিত্তিক অনুশীলন যুক্ত করা যায় | Ich weiß nicht, wie ich evidenzbasierte Verfahren in meine Tätigkeit integrieren kann |
| ***Job-security*** | | |
| Learning an evidence-based practice will help me keep my job | প্রমাণ-ভিত্তিক অনুশীলন সম্পর্কে শিক্ষা আমাকে আমার কাজ ধরে রাখতে সাহায্য করবে | Das Aneignen eines evidenzbasierten Verfahrens hilft mir, meinen Arbeitsplatz zu sichern |
| Learning an evidence-based practice will help me get a new job | প্রমাণ ভিত্তিক অনুশীলন সম্পর্কে শিক্ষা আমাকে একটি নতুন কাজ পেতে সাহায্য করবে | Das Aneignen eines evidenzbasierten Verfahrens hilft mir, einen neuen Job zu finden |
| Learning an evidence-based practice will make it easier to find work | প্রমাণ-ভিত্তিক অনুশীলন সম্পর্কে শিক্ষা কাজটি সহজ করে তুলবে | Das Aneignen eines evidenzbasierten Verfahrens erleichtert es, Arbeit zu finden |
| ***Limitations*** | | |
| Evidence-based practice is not useful for clients with multiple problems | প্রমাণ-ভিত্তিক অনুশীলন একাধিক সমস্যায় আক্রান্ত ক্লায়েন্টদের জন্য উপযোগী নয় | Evidenzbasierte Praxis ist nicht hilfreich für Patienten mit multiplen Störungen |
| Evidence-based practice is not individualized treatment | প্রমাণ-ভিত্তিক অনুশীলন স্বতন্ত্র চিকিৎসা নয় | Evidenzbasierte Praxis ist keine individualisierte Behandlung |
| Evidence-based practice is not narrowly focused | প্রমাণ ভিত্তিক অনুশীলন সংকীর্ণভাবে উপস্থাপন করা হয় না | Evidenzbasierte Praxis ist nicht eng umgrenzt |
|  |  |  |
| If you received training in a therapy or intervention that was new to you, how likely would you adopt it if | যদি আপনি কোন নতুন থেরাপি বা হস্তক্ষেপের প্রশিক্ষণ পান তবে তা আপনি কিভাবে পেয়েছেন তা নিচের প্রশ্নগুলির মাধ্যমে প্রকাশ করুন | Für die folgenden Fragen nehmen Sie bitte an, dass Sie ein Training für ein manualisiertes, evidenzbasiertes Behandlungsverfahren erhalten, das Ihnen noch nicht vertraut ist |
| ***Appeal*** | | |
| it “made sense” to you? | এটা আপনার কাছে "অর্থপূর্ণ"? | es Ihnen sinnvoll erscheint? |
| it was being used by colleagues who were happy with it? | যেসব সহকর্মী এটার প্রতি খুশি ছিল তাঁদের দ্বারা এটা ব্যবহৃত? | es von Kolleg*innen genutzt wird, die damit zufrieden waren? |
| you felt you had enough training to use it correctly? | আপনি কি অনুভব করেন যে সঠিকভাবে এটি ব্যবহার করার জন্য আপনার যথেষ্ট প্রশিক্ষণ ছিল? | Sie das Gefühl haben, Sie wurden ausreichend geschult, um es adäquat anzuwenden? |
| ***Fit*** |  |  |
| you knew it was right for your clients? | আপনি জানেন কি এটা আপনার ক্লায়েন্টদের জন্য সঠিক ছিল? | Sie wüssten, dass die Behandlung das Richtige für Ihre Patient*innen wäre? |
| you had a say in how you would use the evidence-based practice? | আপনি কিভাবে বললেন যে আপনি প্রমাণ-ভিত্তিক অনুশীলন ব্যবহার করবেন? | Sie mitbestimmen dürften, wie Sie das evidenzbasierte Verfahren anwenden? |
| it fit with your clinical approach? | এটা কি আপনার ক্লিনিকাল পদ্ধতির সঙ্গে মানানসই? | es Ihrer üblichen Herangehensweise entsprechen würde? |
| ***Requirements*** |  |  |
| it was required by your supervisor? | এটা আপনার সুপারভাইজার দ্বারা অনুমোদিত ছিল? | Ihr/e Vorgesetzte/r es verlangen würde? |
| it was required by your agency/organization? | এটা আপনার সংস্থা / প্রতিষ্ঠানের দ্বারা অনুমোদিত ছিল? | es an Ihrem Arbeitsplatz verlangt würde? |
| it was required by your state? | এটা আপনার রাষ্ট্র দ্বারা অনুমোদিত ছিল? | wenn es in Ihrem Bundesland vorgesehen wäre? |

**Barriers and Facilitators Scale**

| **English version** | **Bangla version** | **German version** |
| --- | --- | --- |
| The current policy and legislative framework in Bangladesh enables me to use EBPs | বাংলাদেশে বর্তমান নীতি ও আইনী কাঠামো আমাকে প্রমাণ-ভিত্তিক অনুশীলন ব্যবহার করতে সহায়তা করে | Die aktuelle Politik und Gesetzgebung ermöglichen die Anwendung von EBPs |
| My organization’s goals and objectives support the usage of EBPs | আমার প্রতিষ্ঠানের লক্ষ্য ও উদ্দেশ্য প্রমাণ-ভিত্তিক অনুশীলন ব্যবহার করার সমর্থন দেয় | Die Ziele meiner Organisation unterstützen die Anwendung von EBPs |
| The economic climate nationally and locally is conducive to implement and use EBPs | অর্থনৈতিক জলবায়ু জাতীয় এবং স্থানীয়ভাবে প্রমাণ-ভিত্তিক অনুশীলন বাস্তবায়ন ও ব্যবহার করার জন্য সহায়ক | Das wirtschaftliche Klima auf nationaler und lokaler Ebene ist für die Implementierung  und Anwendung von EBPs förderlich |
| I have access to literature on EBPs (e.g. clinical or academic journals) | আমার প্রমাণ-ভিত্তিক অনুশীলনের উপর সাহিত্যের সহজলভ্যতা আছে (উদাঃ ক্লিনিকাল বা একাডেমিক জার্নাল) | Ich habe Zugang zu Literatur über EBPs (z.B. klinische oder akademische Zeitschriften) |
| I have been informed early on the usage of EBPs at my organization | আমার প্রতিষ্ঠান আমাকে প্রমাণ-ভিত্তিক অনুশীলন ব্যবহার করার বিষয়ে প্রাথমিকভাবে তথ্য প্রদান করেছে | Ich werde frühzeitig über die Anwendung neuer EBPs an meinem Arbeitsplatz informiert |
| I have been involved in the implementation of EBPs | আমি প্রমাণ-ভিত্তিক অনুশীলনগুলো বাস্তবায়নের সাথে সম্পৃক্ত | Ich werde an der Implementierung von EBPs beteiligt |
| Key stakeholders at my organization have been informed early on EBPs | আমার প্রতিষ্ঠানের মূল অংশীদারদের প্রমাণ-ভিত্তিক অনুশীলন সম্পর্কে আগে থেকেই অবগত করা হয়েছে | Interessensvertreter*innen in der Organisation werden frühzeitig über Neuerungen  informiert |
| Key stakeholders have been involved in the implementation of EBPs | মূল অংশীদাররা প্রমাণ-ভিত্তিক অনুশীলন  বাস্তবায়নের সাথে সম্পৃক্ত | Interessenvertreter*innen werden an der Implementierung von EBP beteiligt |
| The necessary resources (e.g. funding, adequate staff with appropriate skills, training  and ongoing support) for using EBPs are available where I work | আমি যেখানে কাজ করি সেখানে পর্যাপ্ত প্রয়োজনীয় সম্পদ প্রমাণ-ভিত্তিক অনুশীলন ব্যবহার করার জন্য আছে (উদাঃ অর্থায়ন, উপযুক্ত দক্ষতা সম্পন্ন কর্মী, প্রশিক্ষণ এবং চলমান সমর্থন) | Die notwendigen Ressourcen (z.B. Finanzierung, adäquat geschulte Mitarbeiter mit entsprechenden Fertigkeiten, Schulungen) für den Einsatz von EBP sind an meinem Arbeitsplatz verfügbar |
| The existing workflows and structure within my organization supports me in using evidence-based practices | আমার প্রতিষ্ঠানের বিদ্যমান কর্মপ্রবাহ এবং কাঠামো প্রমাণ-ভিত্তিক অনুশীলন ব্যবহার করতে আমাকে সমর্থন করে। | Der bestehende Arbeitsablauf und die Strukturen an meinem Arbeitsplatz sprechen  für die Anwendung von EBPs |
| My supervisors promote the usage of EBPs | আমার সুপারভাইজার আমাকে প্রমাণ-ভিত্তিক অনুশীলন ব্যবহার করতে উৎসাহ দেন। | Die Vorgesetzten unterstützen die Anwendung von EBPs |
| Ongoing training on EBPs is provided to me | প্রমাণ-ভিত্তিক অনুশীলনের উপর চলমান প্রশিক্ষণ দিয়ে আমাকে প্রশিক্ষিত করা হয়। | Fortlaufende EBP-Schulungen werden angeboten |
| I get feedback and support on how to improve my usage of EBPs | আমি কিভাবে প্রমাণ-ভিত্তিক অনুশীলনের ব্যবহার বৃদ্ধি করতে পারি, সে বিষয়ে নিয়মিত প্রতিক্রিয়া এবং সহায়তা পাই। | Ich erhalte Feedback und Unterstützung, um die Anwendung von EBPs zu verbessern |
