# Supplementary material for: Attitudes Toward and Usage of Evidence-Based Mental Health Practices for Autistic Youth in Bangladesh and Germany: A Cross-Cultural Comparison
Source: J Autism Dev Disord. 2024 Jan 26;55(2):635–51. doi: 10.1007/s10803-023-06223-z (PMC11813821; doi:10.1007/s10803-023-06223-z)

**Figure A1**

*Relations between respondent’s age and caseload per year (categorized as few=1-10 patients, moderate = 10-100, and many = more than 100) and openness subscale of the EBPAS-36 for Bangladesh and Germany. Note that age and caseload per year were the only significant predictors of openness.*


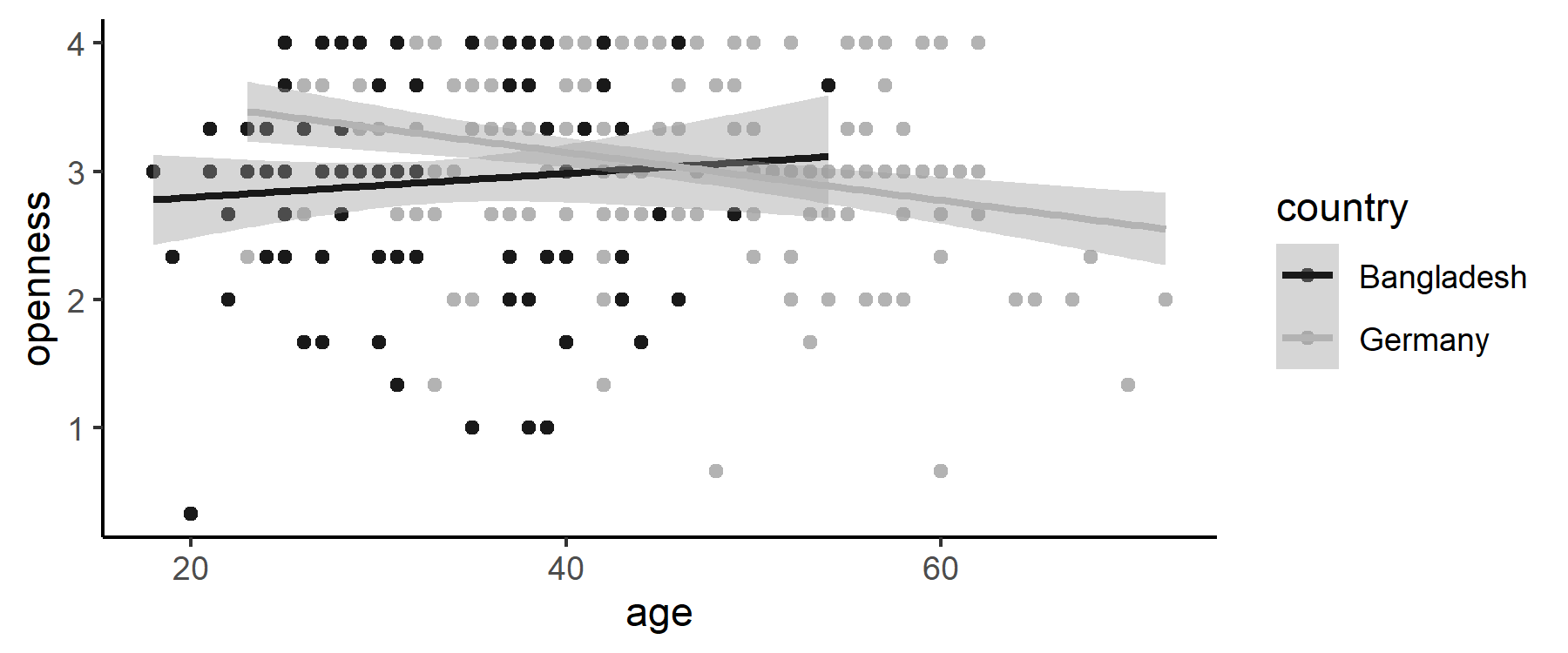


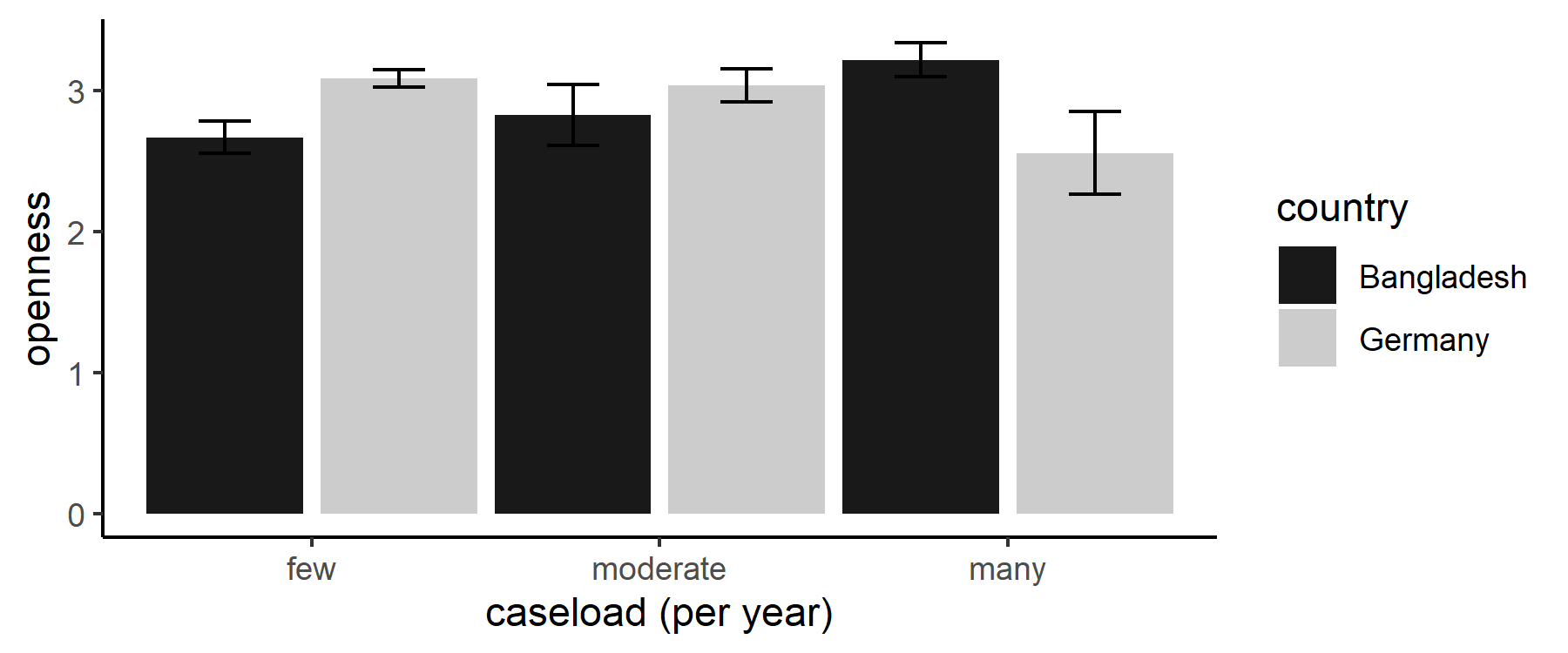


**Figure A2**

*Relation between respondent’s gender (M=male, f=female) and caseload per year (categorized as few=1-10 patients, moderate = 10-100, and many = more than 100) and appeal subscale of the EBPAS-36 for Bangladesh and Germany. Note that gender and caseload per year were the only significant predictors of appeal.*


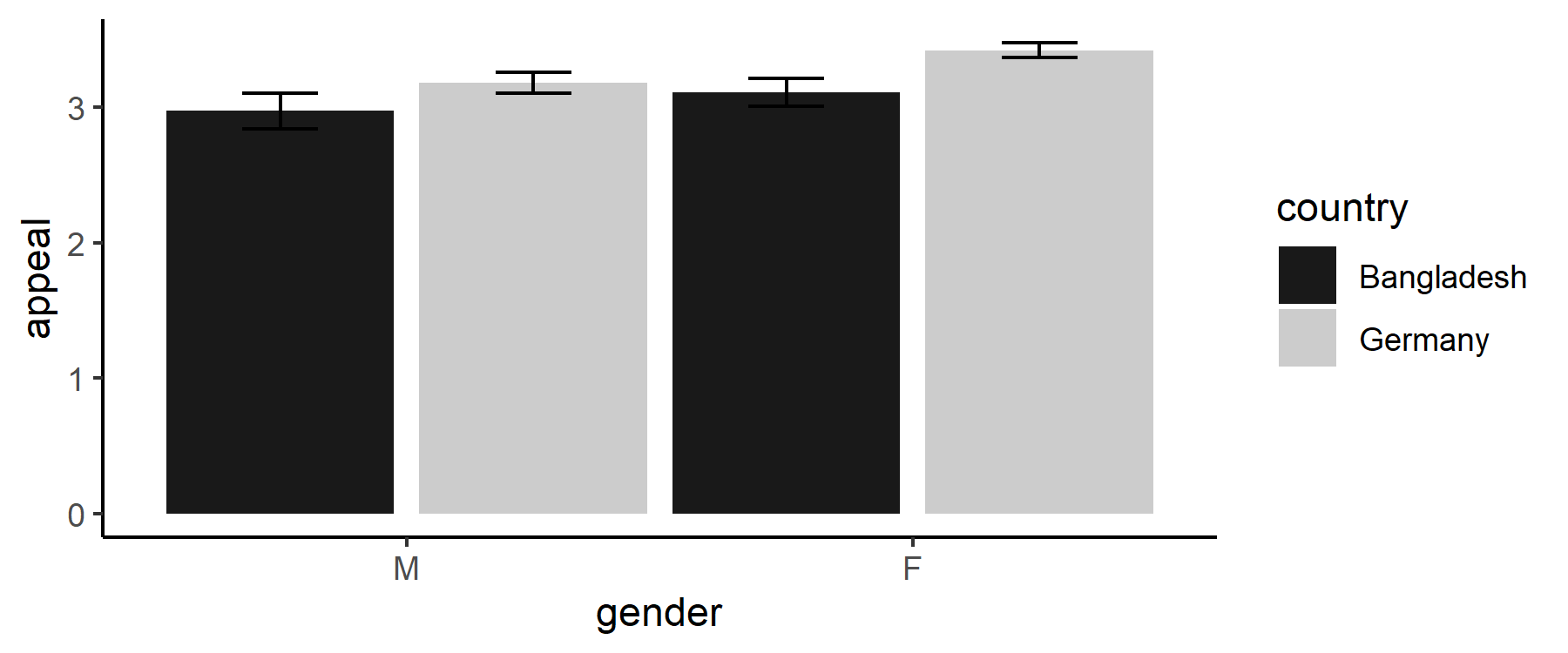


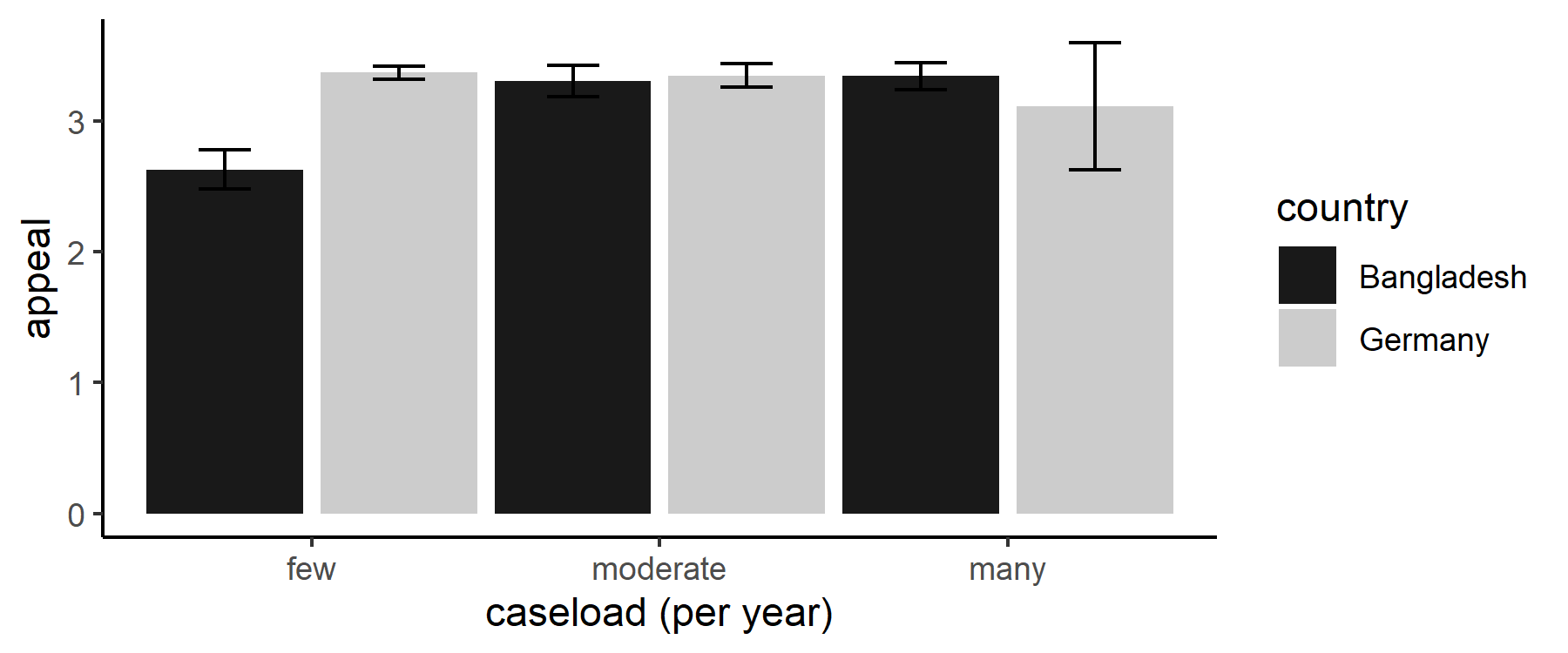


**Figure A3**

*Relation between caseload per year (categorized as few=1-10 patients, moderate = 10-100, and many = more than 100) and working in their own practice (0= no, 1 = yes) and the requirements subscale of the EBPAS-36 for Bangladesh and Germany. Note that caseload per year and working in one’s own practice were the only significant predictors of requirements.*


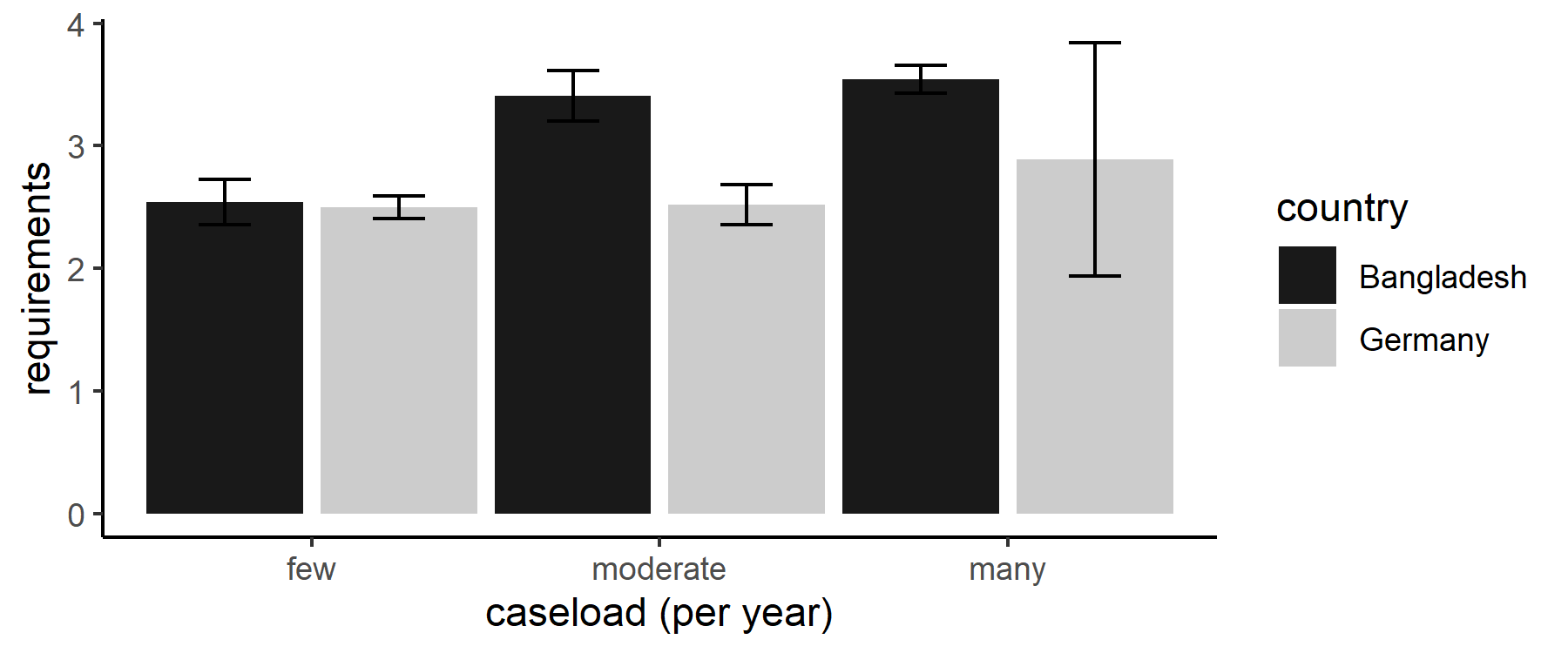


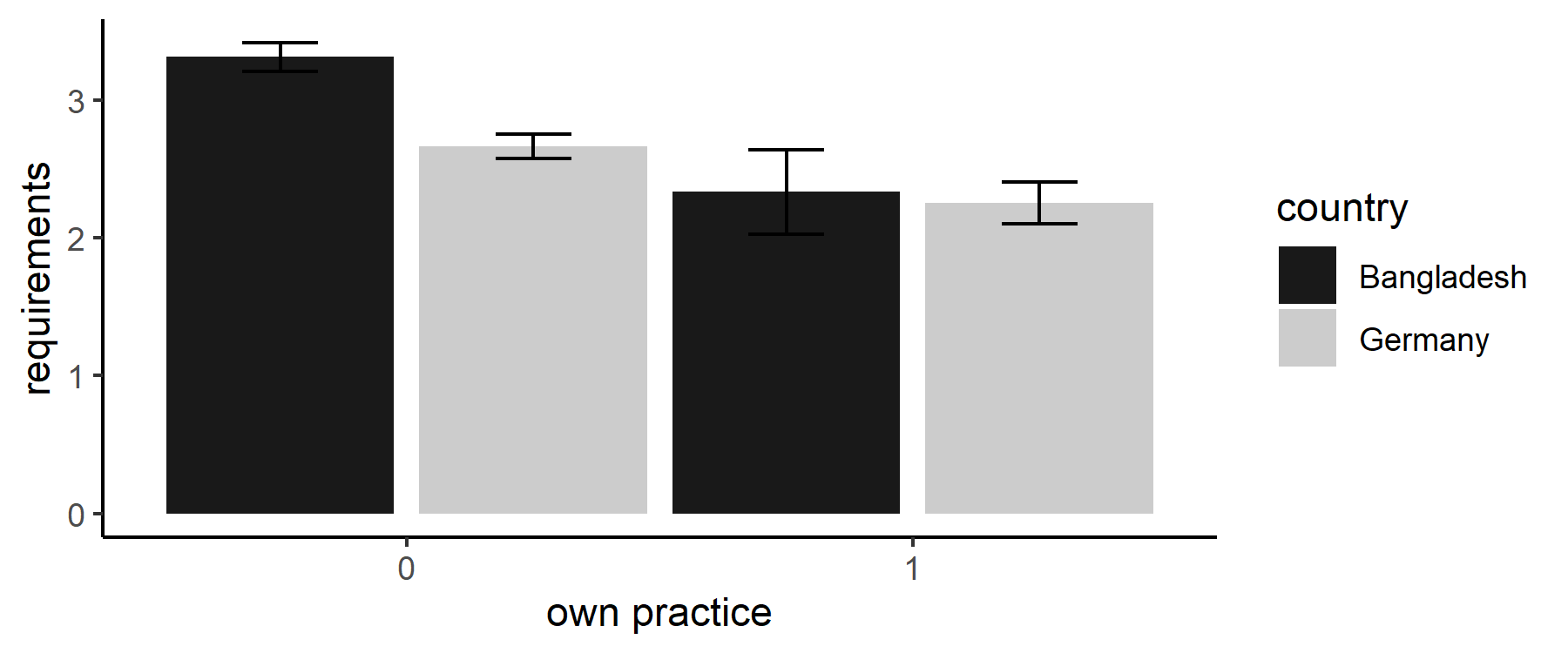


**Figure A4**

*Relation between age and the job security subscale of the EBPAS-36 for Bangladesh and Germany. Note that age was the only significant predictor of job security.*


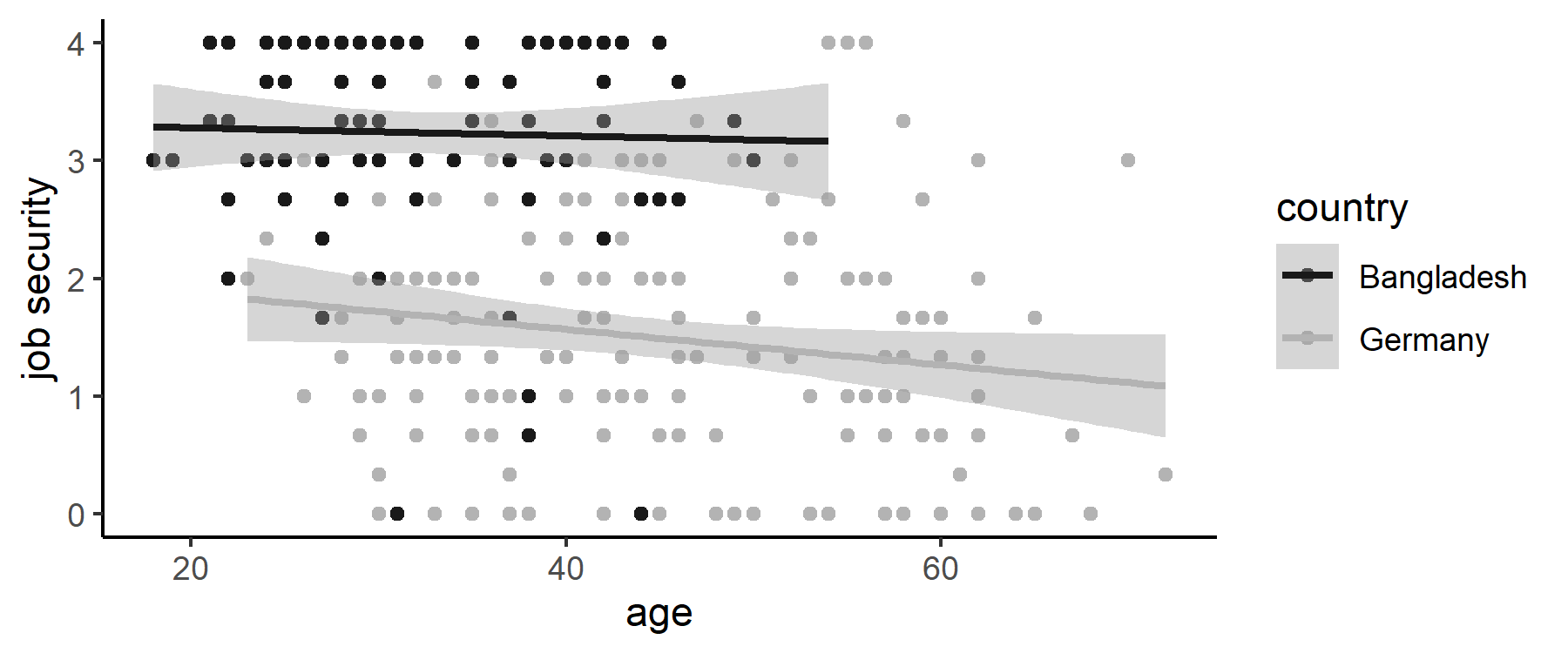


**Figure A5**

*Relation between appeal subscale and the number of different types of EBPs used by professionals in Bangladesh and Germany. Note that appeal subscale is the significant predictor of the number of EBPs used.*


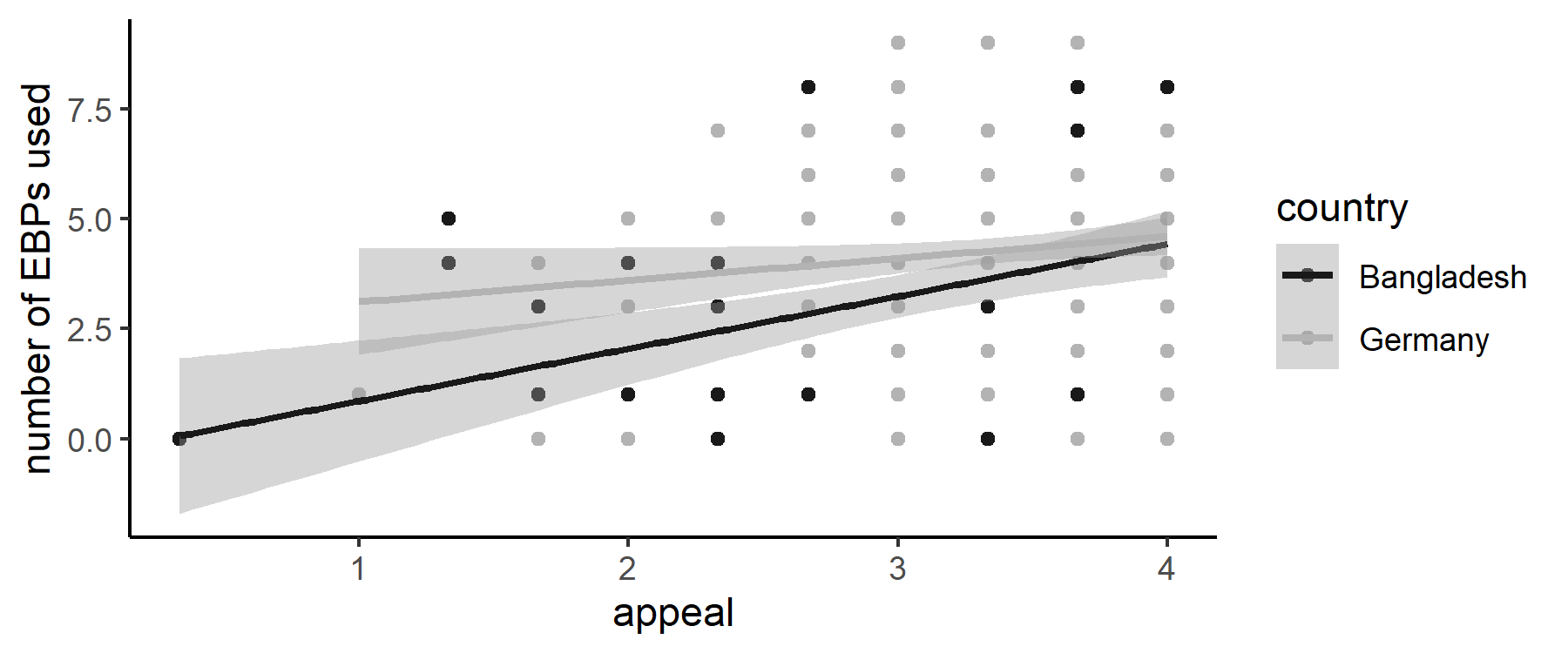

Supplement: Supplementary file 2 — Supplementary file2 (DOCX 25 kb) [file 10803_2023_6223_MOESM2_ESM.docx]
